# A novel method for achieving effective hemostasis using a snare tip positioned within an outer sheath

**DOI:** 10.1055/a-2883-1090

**Published:** 2026-06-12

**Authors:** Haruhiro Inoue, Kohei Shigeta, Kazuki Yamamoto, Ippei Tanaka, Mayo Tanabe, Kei Ushikubo, Yohei Nishikawa

**Affiliations:** 1 Digestive Diseases CenterShowa University Koto Toyosu Hospital, Tokyo, JapanTokyoJapan

## A case description



**Video 1** A novel method for achieving effective hemostasis using a snare tip positioned within an outer sheath.


Snare-tip soft coagulation (STSC) is effective and safe for hemostasis after endoscopic resection of a gastrointestinal tumor. 1​ 2​ ​3​ STSC offers a cost-effective method as no additional equipment is needed. However, adjusting the snare tip to the bleeding point on a two-dimensional screen may be difficult, particularly when close endoscopic access is limited. Currently, a versatile knife (TriangleTipKnife J [TTJ]; Olympus, Tokyo, Japan) with hood attachment (TTJ-H) has provided effective and safe hemostasis as the sheath can be stabilized by pressing it against the gastrointestinal wall. 4​ ​5​ We applied this technique to a snare by cutting the sheath and achieved safe hemostasis.

**Fig. 1:**
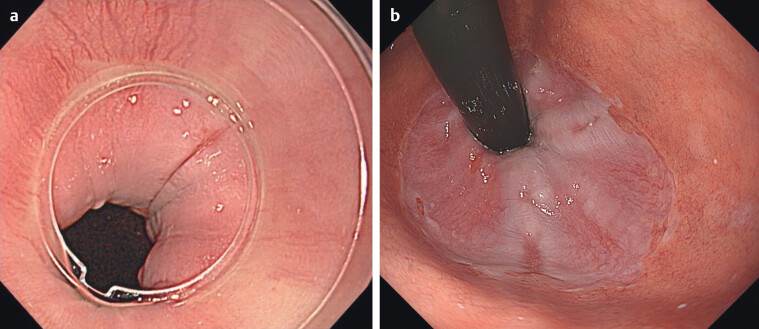
An endoscopic view of the Grade C gastroesophageal reflux disease in a 68-year-old woman with a long-term history of heartburn and chest pain after long-term proton-pump-inhibitor use failed to alleviate her symptoms: ( **a** ) a forward view and ( **b** ) a retroflex view.

**Fig. 2:**
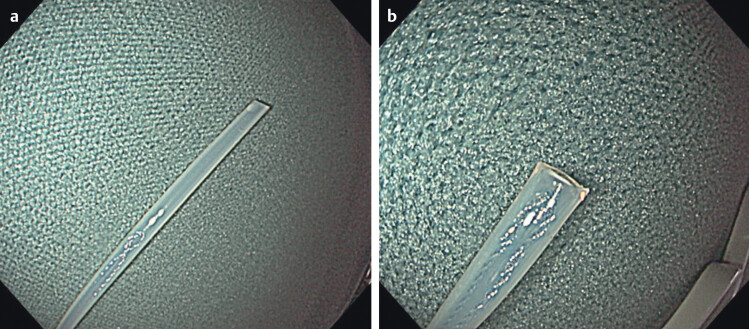
( **a** ) A snare tip positioned within an outer sheath fixing its closed position. ( **b** ) Cutting the snare sheath and eliminated the need for fine endoscopic adjustments.

**Fig. 3:**
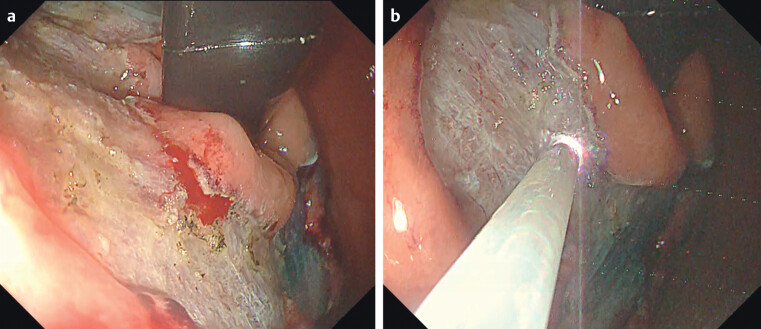
( **a** ) The defect after anti-reflux mucosectomy (ARMS) with oozing. ( **b** ) We obtained effective hemostasis by pushing the snare sheath.

A 68-year-old woman with a long-term history of heartburn was referred to our hospital after long-term proton-pump-inhibitor use failed to alleviate her symptoms, despite ongoing antacid treatment. Upper endoscopy revealed grade B reflux esophagitis (Los Angeles classification; Fig. 1 ), and we performed anti-reflux mucosectomy (ARMS). Cap-assisted endoscopic mucosal resection was performed from the cardia to the upper gastric body along the lesser curvature. After mucosal resection, post-coagulation for exposed vessels and mild oozing on the defect was performed ( Video 1 ). Although STSC was initially attempted, sufficient coagulation was not obtained because of the challenges in distance. Although we attempted TTJ-H–like hemostasis by retracting the snare, the unclear distance between the snare tip and the sheath hindered effective hemostasis. Cutting the snare sheath ( Fig. 2 ) allowed safe hemostasis with spray coagulation mode (ERBE) by pressing it against the defect, similar to the use of the TTJ-H ( **Figs.** 3 **and** 4 ).

**Fig. 4:**
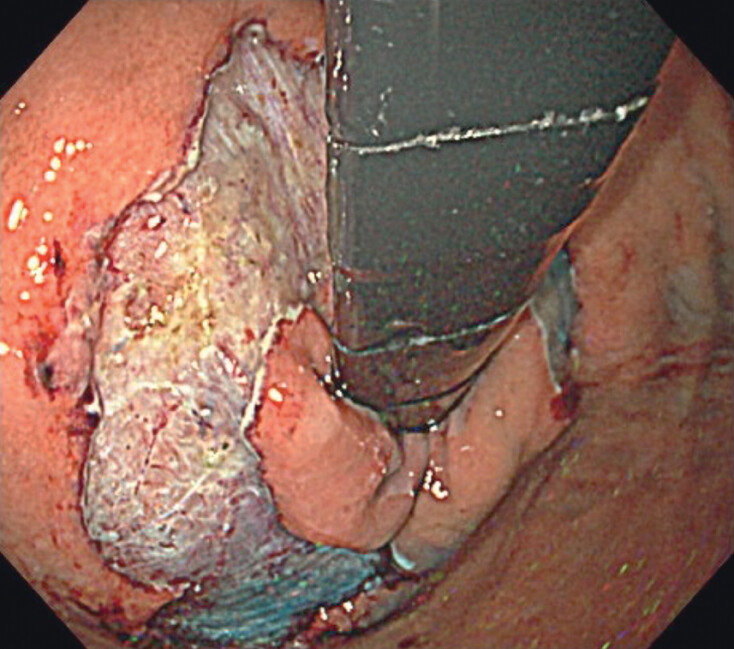
The endoscopic view of the defect after hemostasis.

In this case, fixing its closed position and cutting the snare sheath eliminated the need for fine endoscopic adjustments, allowing safe endoscopic coagulation. Although applied during ARMS, this technique may also be applicable for hemostasis after endoscopic mucosal resection and other bleeding. Given that this method requires neither hemostatic forceps nor argon plasma coagulation, it may offer a more cost-efficient alternative. In summary, this method suggests the potential to provide safe and effective hemostasis.

Endoscopy_UCTN_Code_TTT_1AQ_2A.

## Consent

Informed consent was obtained from the patient to publish the information and images included in this article.
